# A DNA tetrahedron-based ferroptosis-suppressing nanoparticle: superior delivery of curcumin and alleviation of diabetic osteoporosis

**DOI:** 10.1038/s41413-024-00319-7

**Published:** 2024-02-29

**Authors:** Yong Li, Zhengwen Cai, Wenjuan Ma, Long Bai, En Luo, Yunfeng Lin

**Affiliations:** 1 State Key Laboratory of Oral Diseases & National Center for Stomatology & National Clinical Research Center for Oral Diseases, West China Hospital of Stomatology, Sichuan University, Chengdu, Sichuan 610041 PR China; 2 Department of Oral and Maxillofacial Surgery, Affiliated Stomatological Hospital, Southwest Medical University, Luzhou, Sichuan 646000 PR China; 3 Sichuan Provincial Engineering Research Center of Oral Biomaterials, Chengdu, Sichuan 610041 China

**Keywords:** Bone, Osteoporosis

## Abstract

Diabetic osteoporosis (DOP) is a significant complication that poses continuous threat to the bone health of patients with diabetes; however, currently, there are no effective treatment strategies. In patients with diabetes, the increased levels of ferroptosis affect the osteogenic commitment and differentiation of bone mesenchymal stem cells (BMSCs), leading to significant skeletal changes. To address this issue, we aimed to target ferroptosis and propose a novel therapeutic approach for the treatment of DOP. We synthesized ferroptosis-suppressing nanoparticles, which could deliver curcumin, a natural compound, to the bone marrow using tetrahedral framework nucleic acid (tFNA). This delivery system demonstrated excellent curcumin bioavailability and stability, as well as synergistic properties with tFNA. Both in vitro and in vivo experiments revealed that nanoparticles could enhance mitochondrial function by activating the nuclear factor E2-related factor 2 (NRF2)/glutathione peroxidase 4 (GPX4) pathway, inhibiting ferroptosis, promoting the osteogenic differentiation of BMSCs in the diabetic microenvironment, reducing trabecular loss, and increasing bone formation. These findings suggest that curcumin-containing DNA tetrahedron-based ferroptosis-suppressing nanoparticles have a promising potential for the treatment of DOP and other ferroptosis-related diseases.

## Introduction

Diabetic osteoporosis (DOP), a systemic metabolic bone disorder, is the primary cause of reduced bone mass, disruption of bone tissue microstructure, and increased susceptibility to fractures.^1–6^ In 2021, Khosla et al.^7^ reported that patients with diabetes had a 32% higher risk of fractures compared to those without diabetes, with a mortality rate 1.23 times higher than that of individuals with type 2 diabetes alone, leading to a significant burden on patients and the healthcare system. Recently, Hofbauer et al.^8^ proposed that ferroptosis could represent a crucial cellular and molecular mechanisms underpinning the diabetes-induced skeletal changes. Specifically, elevated levels of ferroptosis inhibit the expression of osteogenic transcription factors, such as osterix (OSX; also known as SP7) and Runt-related transcription factor 2 (RUNX2), altering the equilibrium of osteogenic commitment and differentiation of bone mesenchymal stem cells (BMSCs), negatively affecting bone homeostasis.^9,10^ Therefore, targeting ferroptosis presents a new strategy for the treatment of DOP.^9,11–13^

Ferroptosis is a recently identified form of programmed cell death that distinguishes itself from traditional types of programmed cell death, which include apoptosis, autophagy, and pyroptosis,^14,15^ and is characterized by accumulation of iron and lipid peroxidation.^16–18^ Glutathione peroxidase 4 (GPX4), an antioxidative enzyme negatively regulated by endoplasmic reticulum stress and responsible for the elimination of excessive lipid peroxides, is the critical upstream regulator of ferroptosis.^19,20^ Moreover, GPX4 plays a role of the main protection system against ferroptosis in both the cytoplasm and mitochondria.^21^ In the diabetic microenvironment, the intracellular content and activity of GPX4 are significantly reduced, leading to the excessive accumulation of reactive oxygen species (ROS) and Fe^2+^, and the ectopic expression of lipid hydroperoxide and ferroptosis-related proteins, which are considered to be the principal elements that initiate ferroptosis.^22^ The direct application of ferroptosis inhibitor ferrostatin-1 (Fer-1) in the mouse model of DOP could reduce bone loss,^11^ demonstrating that ferroptosis is closely associated with the pathogenesis of DOP. Nuclear factor E2-related factor 2 (NRF2) inhibits ferroptosis by upregulating the expression of GPX4.^23^ Thus, the development of an GPX4 agonist that targets NRF2 activation could effectively eliminate ROS and regulate lipid peroxidation. This approach could inhibit ferroptosis, increase bone formation, and alleviate diabetic osteoporosis.

Curcumin, a polyphenol derived from medicinal plant turmeric, activates the kelch like ECH associated protein 1(KEAP1)/NRF2 pathway.^24^ In addition, curcumin has been extensively studied for its anti-inflammatory, antioxidative, and hypolipidemic properties, as well as for its ability to suppress osteoclastogenesis in vitro.^25–27^ We hypothesized that curcumin targeted the cellular redox metabolism to regulate ferroptosis. However, poor pharmacokinetics, targeting, and toxic side effects of curcumin, when administered repeatedly, hinder its widespread use as a drug.^28^ Therefore, an alternative delivery system is necessary to advance the development of curcumin into a drug.

With the rapid development of nanotechnology, nanomaterials have shown a wide variety of applications in the biomedical field owing to their excellent biosafety, stability, and multifunctional design. Tetrahedral framework nucleic acid (tFNA) can be self-assembled using four single-stranded DNA (ssDNA) molecules via temperature regulation. The unique property enables tFNA to freely traverse cell membranes.^29,30^ It possesses excellent biocompatibility, safety, editability, and stability, making it a DNA nanomaterial with broad potential applications.^31–33^ Prior studies have demonstrated the ability of tFNA to deliver oligonucleotides (e.g., siRNA and microRNA),^34–36^ polypeptides (e.g., OGP),^37^ and natural products (e.g., quercetin and typhaneoside).^38–40^ Moreover, tFNA improves hepatic insulin resistance,^41–43^ reduces blood glucose levels, and alleviates type 2 diabetes (T2DM) through the phosphatidylinositol 3-kinase (PI3K)/Akt pathway.^43^ Therefore, the objective of our study was to synthesize a ferroptosis-suppressing nanoparticle using tFNA that would deliver a natural product, specifically curcumin, to the bone marrow, subsequently resulting in enhanced mitochondrial protection, inhibition of ferroptosis, and induction of BMSC osteogenic differentiation in a diabetic microenvironment. We anticipate our findings to provide information regarding the molecular mechanisms involved in the nanoparticle-mediated inhibition of ferroptosis, identifying a novel strategy for the treatment of DOP.

## Results

### Performance of ferroptosis-suppressing nanoparticle

**Fig. 1:**
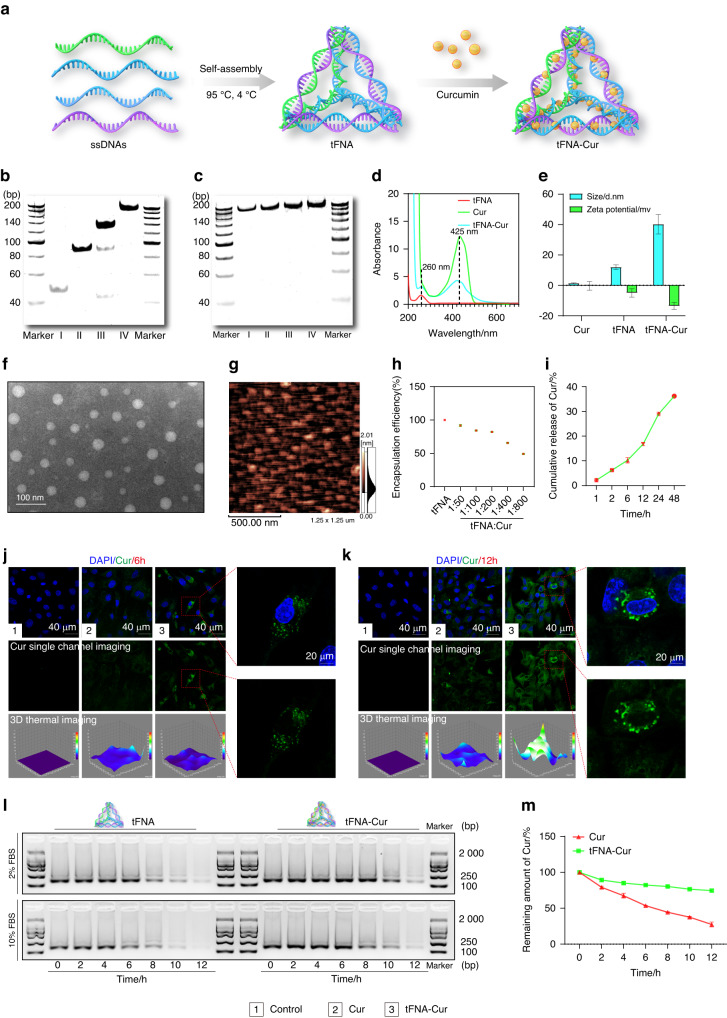
Performance of tFNA-Cur as a ferroptosis-suppressing nanoparticle. **a** Schematic representation of the fabrication of tFNA-Cur. **b** Successful synthesis of tFNA shown in PAGE (I: S1, II: S1 + S2, III: S1 + S2 + S3, IV: tFNA). **c** Successful synthesis of tFNA-Cur depicted in PAGE (I: tFNA, II: tFNA/Cur = 1/50, III: tFNA/Cur = 1/100, IV: tFNA/Cur = 1/200). **d** Absorbance spectra of tFNA (260 nm), curcumin (425 nm), and tFNA-Cur. **e** Molecular size and Zeta potential of tFNA, curcumin, and tFNA-Cur. **f**, **g** TEM and AFM images of the 3D nanostructure of tFNA-Cur. **h** Encapsulation efficiency of curcumin loaded in tFNA. **i** Cumulative release profile of curcumin from tFNA-Cur. **j** Cellular uptake of curcumin and tFNA-Cur observed via IF after 6 h. **k** Cellular uptake of curcumin and tFNA-Cur observed via IF after 12 h. **l** Stability evaluation of tFNA and tFNA-Cur in FBS using PAGE. **m** Stability evaluation of tFNA and tFNA-Cur in PBS. The experimental results are presented as mean ± SD (*n* = 3)

The synthesis of tFNA-Cur involved two steps (Fig. 1a). Initially, tFNA self-assembled through temperature regulation, as demonstrated by polyacryamide gel electrophoresis (PAGE) (Fig. 1b). Subsequently, various concentrations of curcumin (10, 20, and 40 μmol/L) were complexed with tFNA through oscillation for 3 h, and successful encapsulation of curcumin into tFNA was confirmed through PAGE (Fig. 1c). The absorption spectrum of tFNA-Cur in Fig. 1d also confirmed this result. The size of tFNA was measured at 12.03 ± 1.499 nm, and tFNA-Cur was 40.23 ± 6.41 nm, with zeta potentials of −4.93 ± 2.74 mV and −13.5 ± 2.28 mV, respectively (Fig. 1e, Fig. S1b, c). TEM and AFM further demonstrated that the size of tFNA-Cur was 40-50 nm, maintaining the 3D nanostructure of tFNA (Fig. 1f, g).

The encapsulation efficiency and curcumin release from tFNA-Cur were determined by evaluating the proportion of curcumin carried by tFNA. With a fixed tFNA concentration of 200 nmol/L, various concentrations of curcumin (10, 20, 40, 80, and 160 μmol/L) were encapsulatedna into tFNA, resulting in a decreasing curve for encapsulation efficiency. The encapsulation efficiencies of curcumin at 10, 20, and 40 μmol/L were found to be 91.825 9%, 84.288 1%, and 82.226%, respectively (Fig. 1h). The tFNA exhibited excellent drug-loading capacity within 40 μmol/L. Moreover, curcumin showed sustained release from tFNA-Cur over 48 h, remaining at 40%, which is significant for clinical applications (Fig. 1i). The excellent water solubility of tFNA-Cur was confirmed by the appearance of synthesized curcumin and tFNA-Cur (Fig. S1a). As a result, we have successfully fabricated a ferroptosis-suppressing nanoparticle, known as “tFNA-Cur,” with a simple synthesis process, excellent drug-loading capacity, and high utilization ratio of the drug.

The issues of poor bioavailability and instability in physiological fluids that significantly restrict the clinical application of natural products are addressed by tFNA-Cur. We investigated the cellular uptake of curcumin and tFNA-Cur using confocal microscopy. The immunofluorescence (IF) images (Fig. 1j, k) revealed that the uptake of tFNA-Cur was superior to that of curcumin at both 6 h and 12 h, with the best penetration effect observed at 12 h, further demonstrating the excellent delivery function of tFNA-Cur. We validated the serum stability of tFNA-Cur (Fig. 1l, m). Dissociative curcumin rapidly degraded in TM buffer within 2 h, with the residual amount reaching only 27.459% at 12 h, significantly weaker than the 74.566 5% of tFNA-Cur. Moreover, PAGE showed that tFNA-Cur could stably exist in the serum for 12 h, which is longer than dissociative tFNA. The results at 2% and 10% were consistent. Finally, we explored the stability of tFNA and tFNA-Cur labeled with Cy5 in mice and found that tFNA-Cur remained in physiological fluids for 24 h, superior to the stability of dissociative tFNA (Fig. S2). In conclusion, the synthesized ferroptosis-suppressing nanoparticle has successfully addressed the bottleneck of poor bioavailability and instability of natural products in physiological fluids, making it highly promising for a wide range of applications.

### Establishment of DOP model and exploration of the ferroptosis in vivo

**Fig. 2:**
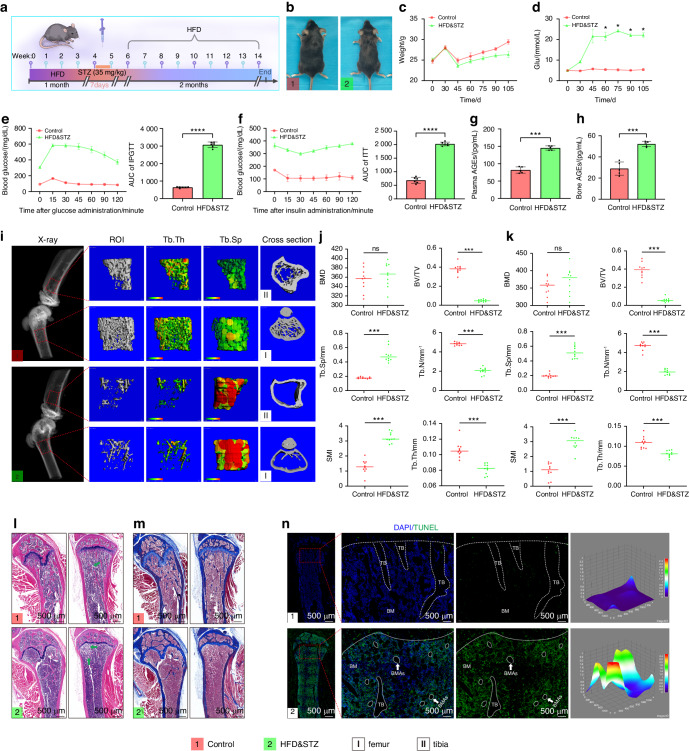
Establishment of a model of diabetic osteoporosis and exploration of ferroptosis in vivo. **a** Schematic representation of the establishment of DOP. **b** Images of Control and HFD&STZ mice. **c** Biweekly assessment of body weight. **d** Biweekly measurement of blood glucose levels. **e**, **f** IPGTT and **g**, **h** ITT performed of Control and HFD&STZ. **i** Representative X-ray and micro-CT images showing the region 2 mm below the epiphyseal of the femur (I) and tibia (II) (Scale bar: 100 μm). **j**, **k** Quantitative analysis of bone parameters using micro-CT (BMD, BV/TV, Tb.N, Tb.Th, Tb.Sp, and SMI) (Right: femur, Left: tibia). **l**, **m** H&E and masson staining of bone tissues (Left: femur, Right: tibia, Scale bar: 500 μm, Arrow: BMAs). **n** Representative images of the TUNEL assay (Nucleus: blue, TUNEL-positive cells: green, Scale bar: 50 μm, BM: bone marrow, TB: femur trabeculae, Circle: BMAs). The experimental results are presented as mean ± SD (*n* = 6). *P < 0.05; **P < 0.01; ***P < 0.001

We established a model of DOP using a high-fat diet (HFD) and low-dose streptozotocin (STZ) (Fig. 2a). Biweekly estimations of body weight and plasma glucose levels were conducted, and inraperitoneal glucose tolerance test (IPGTT) and insulin tolerance test (ITT) were performed on the day of euthanasia. The plasma glucose levels in the HFD&STZ mice continued to rise, while their weights decreased (Fig. 2b–d). IPGTT and ITT demonstrated impaired glucose tolerance and insulin resistance in the HFD&STZ mice (Fig. 2e, f). Pancreatic sections also supported this result (Fig. S3). Interestingly, we found the levels of advanced glycation end products (AGEs) in the plasma and bone of HFD&STZ mice also increased (Fig. 2g, h). AGEs are known markers of diabetes, and their accumulation leads to various complications.^44–46^ These findings indicate that AGEs accumulation is important characteristics of the diabetic microenvironment.

We found that HFD&STZ mice exhibited severe trabecular breakage, absorption, and osteopenia, similar to observations in osteoporosis. Quantitative analysis revealed that the bone volume/total volume ratio (BV/TV), trabecular number (Tb. N), and trabecular thickness (Tb. Th) of HFD&STZ mice decreased, whereas trabecular separation (Tb. SP) and skeletal muscle mass index (SMI) increased. Interestingly, bone mineral density (BMD) between the HFD&STZ and control groups did not show significant differences, consistent with previous studies (Fig. 2i–k). Furthermore, we evaluated the bone microstructure changes in the model mice using hematoxylin and eosin (H&E) and masson staining, and the results are consistent with the micro-CT (Fig. 2l, m).

To further explore the correlation between osteogenesis and ferroptosis in the diabetic microenvironment, we performed TdT-mediated dUTP nick-end labeling (TUNEL) of tibial. The results of TUNEL staining showed a significant increase in apoptotic cells in the modeling group (Fig. 2n, Fig. S4). We successfully constructed a model of DOP and showed that ferroptosis is closely associated with the pathogenesis of DOP.

### Increased osteogenic potential of BMSCs in diabetic microenvironment by tFNA-Cur

**Fig. 3:**
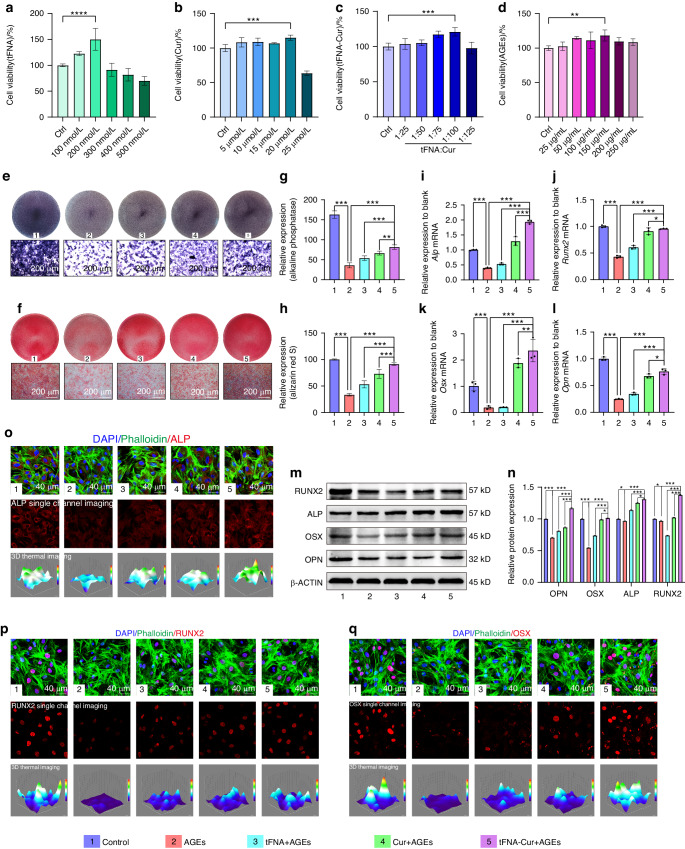
Enhanced osteogenic potential of BMSCs in diabetic microenvironment by tFNA-Cur. **a–c** BMSCs were treated with different concentrations of tFNA, curcumin, and tFNA-Cur for 12 h, and cell viability was assessed using the CCK-8 kit. **d** BMSCs were treated with AGEs at various concentrations for 24 h, and cell viability was evaluated using the CCK-8 kit. **e**, **f** Osteogenic differentiation was observed through Alkaline Phosphatase and Alizarin Red staining. **g**, **h** Quantitative analysis of ALP and Alizarin Red staining. **i–l** Relative gene expression levels of *Alp*, *Runx2*, *Osx*, and *Opn* analyzed by RT-PCR. **m,** **n** WB results and quantitative analysis of ALP, RUNX2, OSX, and OPN protein expression levels. **o** IF analysis of ALP expression in BMSCs after different treatments. **p** IF analysis of RUNX2 expression in BMSCs after different treatments. **q** IF analysis of OSX expression in BMSCs after different treatments. The experimental results are presented as mean ± SD (*n* = 3). Statistical analysis: *P < 0.05, **P < 0.01, and ***P < 0.001

The osteogenic potential of BMSCs, crucial osteoblastic precursor cells in the bone marrow stroma,^47,48^ is inhibited within the diabetic microenvironment. Thus, enhancing the osteogenic potential of BMSCs is crucial for treating DOP. Initially, BMSCs were isolated, cultivated, and identified (Fig. S5a–c). Subsequently, BMSCs were treated with tFNA-Cur or AGEs to assess cell viability. Notably, tFNA-Cur exhibited a concentration-dependent effect on BMSCs, with optimal cell activity observed at tFNA 200 nmol/L and curcumin 20 μmol/L (Fig. 3a–c). Different concentrations of AGEs were tested, and 150 μg/mL was chosen for subsequent experiments since it inhibited the osteogenic potential of BMSCs while maintaining high cell viability (Fig. 3d, Fig. S6a, b).

We treated BMSCs with AGEs to simulate a diabetic microenvironment and investigated the biological functions of tFNA-Cur. As depicted in Fig. 3e, f, osteogenic differentiation and calcium nodule formation significantly decreased in the diabetic microenvironment. However, the tFNA, curcumin, and tFNA-Cur pretreatment groups demonstrated improvement, particularly the tFNA-Cur group (Fig. 3g, h). Additionally, we assessed the expression of *Alp, Runx2, Osx*, and *Opn* through RT-PCR, which were substantially inhibited in the treatment group but reversed by tFNA-Cur pretreatment (Fig. 3i–l). These findings were further supported by protein level analyses (Fig. 3m–q, Fig. S7a–c). ALP (a marker for osteoblasts), OSX (a specific marker for osteoblast progenitor cells) and RUNX2 (a marker for mature osteoblasts) were determined using western blot (WB) and immunofluorescence. Consistent patterns revealed that tFNA-Cur significantly promoted osteogenesis in the diabetic microenvironment compared to tFNA or curcumin alone.

### tFNA-Cur suppresses AGEs-induced ferroptosis via NRF2/GPX4 pathway

**Fig. 4:**
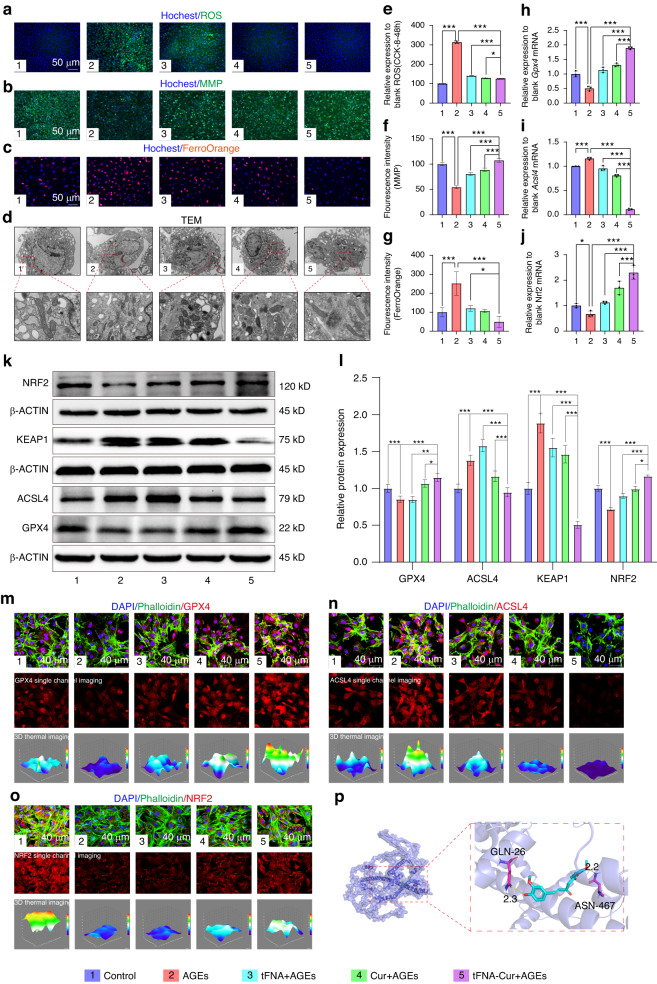
tFNA-Cur Suppresses AGEs-Induced Ferroptosis via the NRF2/GPX4 Pathway. **a–d** Representative images of ROS staining (Nucleus: blue; ROS: Green, Scale bar: 50 μm), MMP staining (Nucleus: blue; MMP: Green, Scale bar: 50 μm), FerroOrange staining (Nucleus: blue; Fe^2+^: Orange, Scale bar: 50 μm), and TEM images (Scale bar: 1 μm and 200 nm) after treating BMSCs with tFNA, curcumin, and tFNA-Cur for 12 h, followed by AGEs for 24 h. **e**–**g** Quantitative analysis of ROS, MMP, and Fe^2+^ levels. **h–j** RT-PCR analysis of *Gpx4*, *Acsl4*, and *Nrf2* expression. **k**, **l** WB analysis and quantitative assessment of ACSL4, GPX4, NRF2, and KEAP1 expression levels. **m** IF analysis of GPX4 expression in BMSCs after different treatments. **n** IF analysis of ACSL4 expression in BMSCs after different treatments. **o** IF analysis of NRF2 expression in BMSCs after different treatments. **p** 3D docking simulation of NRF2 and Curcumin (NRF2: dark blue, Curcumin: cyan, Binding points: magenta). The experimental results are presented as mean ± SD (*n* = 3). Statistical analysis: *P < 0.05, **P < 0.01, and ***P < 0.001

Ferroptosis is characterized by lipid peroxidation, iron-dependent accumulation, and downregulation of GPX4. Morphologically, shrunken mitochondria, densely condensed mitochondria, and reduced or vanished mitochondrial cristae are unique features of ferroptosis. To assess changes in reactive oxygen species (ROS) and mitochondrial membrane potential (MMP), we treated cells with AGEs and tFNA-Cur, then observed them using fluorescence microscopy. AGEs stimulation resulted in significant ROS accumulation and mitochondrial membrane rupture, whereas tFNA and curcumin treatment suppressed ferroptosis, with tFNA-Cur showing a more pronounced reduction in ROS levels and membrane damage (Fig. 4a, b, e, f). Similar results were obtained using FerroOrange staining, which indicated iron-dependent accumulation in BMSCs when treated with AGEs. However, the tFNA-Cur pretreatment group exhibited significantly lower Fe^2+^ levels in the diabetic microenvironment (Fig. 4c, g). Mitochondrial morphology is a crucial indicator of ferroptosis, characterized by shrunken and densely condensed mitochondria with reduced or vanished cristae. Using TEM, we evaluated mitochondrial changes in the diabetic microenvironment. While AGEs induced mitochondrial alterations, tFNA-Cur pretreatment restored mitochondria to a normal state more effectively than tFNA and curcumin alone (Fig. 4d). Western blot and immunofluorescence were used to detect the levels of GPX4 and ACSL4. tFNA-Cur treatment significantly decreased GPX4 expression, however, the trend for ACSL4 was the opposite (Fig. 4k–n, Fig. S7d, e). These findings were also supported by gene expression levels (Fig. 4h, i). tFNA-Cur can significantly enhance mitochondrial defense (GPX4 pathway), protecting BMSCs against ferroptosis.

Combined with the results from the mouse model, ferroptosis of BMSCs inhibits their osteogenic potential in the diabetic microenvironment. Therefore, targeting ferroptosis could be an effective method for treating DOP. In 2023, Liang et al. reported five known ferroptosis suppressor genes: *Slc7a11*, *Nrf2*, *Fsp1*, *Gch1*, and *Nos2*.^23^ We hypothesize that NRF2 could be a target gene for DOP treatment, as it upregulates GPX4 expression, leading to ferroptosis inhibition. We employed AlphaFold and AutoDock Vina (version 1.2.0) to predict the protein structure of NRF2 and its molecular docking with Curcumin.^49–51^ The interaction analysis (Fig. 4p) revealed multiple groups of interaction forces (Table S4), such as a hydrogen bond between Asp408 of NRF2 and curcumin, with a binding energy of -7.1 kcal/mol. Based on these findings and previous studies, we propose that curcumin could inhibit ferroptosis by activating the NRF2/GPX4 pathway.

Molecular docking results indicated NRF2 as the target of tFNA-Cur for inhibiting ferroptosis. Accordingly, we assessed NRF2 expression using western blot and immunofluorescence (Fig. 4j–l, o, Fig. S7f), and as expected, similar to GPX4, NRF2 activation was observed upon tFNA-Cur treatment. In conclusion, tFNA-Cur inhibits ferroptosis through the NRF2/GPX4 pathway in the diabetic microenvironment.

### Targeting ferroptosis alleviates DOP using tFNA-Cur

**Fig. 5:**
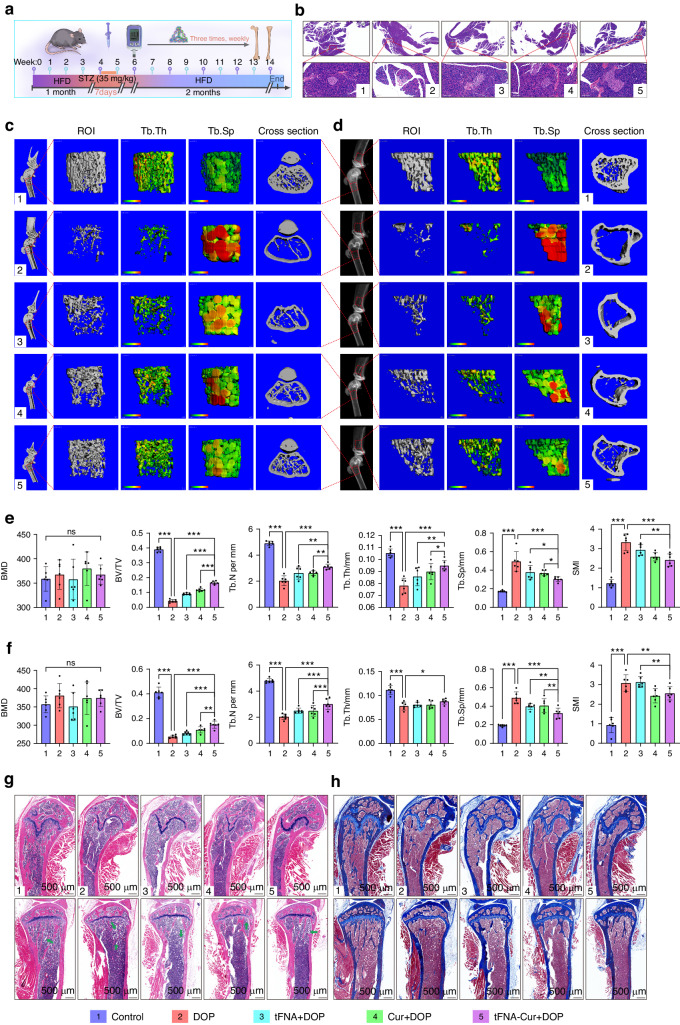
Alleviation of diabetes osteoporosis by tFNA-Cur targeting ferroptosis in vivo. **a** Administration of tFNA, curcumin, and tFNA-Cur for the in vivo treatment of DOP. **b** H&E staining of pancreatic tissue (Scale bar: 1 mm and 100 µm). **c**, **d** Representative X-ray and micro-CT images of the femur (right) and tibia (left) taken 2 mm below the epiphysis. **e**, **f** Quantitative analysis of bone parameters using micro-CT (BMD, BV/TV, Tb.N, Tb.Th, Tb.Sp, and SMI) (Upper: femur, Below: tibia). **g**, **h** H&E and masson staining of the femur (upper) and tibia (below) (Scale bar: 1 mm, Arrow: BMAs). The experimental results are presented as mean ± SD (*n* = 6). Statistical analysis: *P < 0.05, **P < 0.01*,* and ***P < 0.001

To demonstrate our findings in both animal and in vitro experiments, we intraperitoneally injected equal volumes of tFNA, curcumin, and tFNA-Cur into DOP mice (Fig. 5a). tFNA-Cur exhibited superior effects in reducing plasma sugar and AGEs compared to tFNA and curcumin alone (Fig. S8a–c). This enhanced effect may be attributed to the protective impact of tFNA-Cur on pancreatic islets (Fig. 5b). Moreover, the results presented in Fig. 5c–f illustrate that tFNA-Cur treatment significantly restored trabecular integrity and effectively reduced bone loss when compared to the other treatment groups. These findings were further supported by H&E and masson staining, revealing a substantial increase in the abundance of trabeculae and collagen fibers (Fig. 5g, h). Interestingly, DOP mice exhibited increased accumulation of bone marrow adipocytes (BMAs), consistent with prior research (Fig. S9a–c).^4^ Remarkably, tFNA-Cur exhibited no toxicity in mice and effectively treated complications arising from diabetes, as shown in Fig. S10 for the Lung, Liver, and Kidney.

**Fig. 6:**
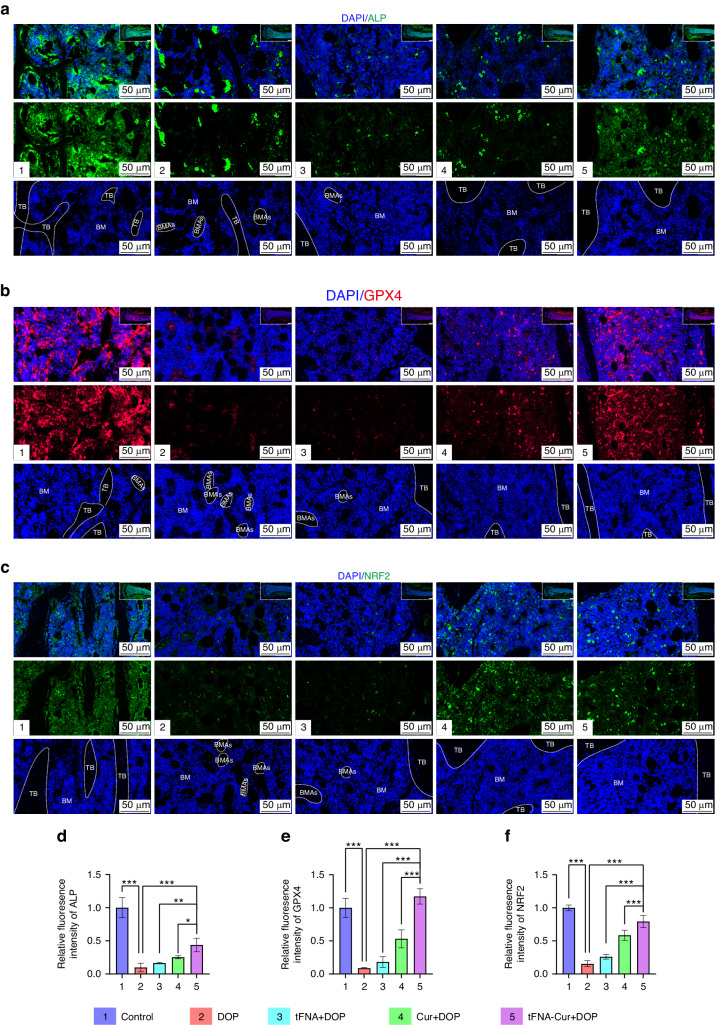
Enhancement of osteogenesis by tFNA-Cur through the NRF2/GPX4 pathway in vivo. **a** IF analysis of ALP expression in bone tissues (Nucleus: blue, ALP: green, Scale bar: 50 µm, BM bone marrow, TB femur trabeculae, BMAs bone marrow adipocytes). **b** IF analysis of GPX4 expression in bone tissues (Nucleus: blue, GPX4: red, Scale bar: 50 µm, BM bone marrow, TB femur trabeculae, BMAs bone marrow adipocytes). **c** IF analysis of NRF2 expression in bone tissues (Nucleus: blue, NRF2: green, Scale bar: 50 µm, BM bone marrow, TB femur trabeculae, BMAs bone marrow adipocytes). **d–f** Quantitative analysis of ALP, GPX4, and NRF2 expression in vivo based on IF staining. The experimental results are presented as mean ± SD (*n* = 3). Statistical analysis: *P < 0.05, **P < 0.01, and ***P < 0.001

In addition to its positive effects on trabecular homeostasis, tFNA-Cur significantly inhibited ferroptosis and activated the osteogenic potential of BMSCs. To elucidate the underlying mechanism, we examined the molecular expression of ALP, GPX4, and NRF2 in the different treatment groups. tFNA-Cur significantly promoted the expression of NRF2, thereby activating the antioxidant defense system (Fig. 6c, f), and upregulated the expression of GPX4, effectively enhancing mitochondrial defense and inhibiting ferroptosis (Fig. 6b, e). Ultimately, tFNA-Cur exerted its inhibitory effects on ferroptosis through the NRF2/GPX4 pathway, thereby activating the osteogenic potential of BMSCs in the diabetic microenvironment (Fig. 6a, d).

## Discussion

Ferroptosis was first identified in 2012 and is described as a unique iron-dependent type of cell death.^14,19,21^ Excessive osteocyte death has been observed in the diabetic bone due to the presence of apparent ferroptosis phenotype. This phenotype includes lipid peroxide accumulation, morphological alterations of the mitochondria, iron overload, and ferritin gene activation. These findings indicate that ferroptosis is a target for the treatment of DOP.^9,11^

DOP is a type of secondary osteoporosis, which could be divided into type 1 and type 2.^8^ Compared with type 1, type 2 has a higher morbidity and increased bone microstructure damage. In this study, we constructed a type 2 DOP mouse model using HFD and continuous low-dose STZ. Consistent with other reports,^1,7^ no significant differences were observed in bone mineral density (BMD) in type 2 DOP mice; however, their bone volume/total volume ratio (BV/TV), trabecular number (Tb. N), and trabecular thickness (Tb. Th) were significantly reduced compared to that in the control group. One possible explanation is that two-month timeline was insufficient for cortical bone remodeling. Further, we observed that cell death and AGE accumulation in the bones of type 2 DOP mice were increased compared to those in the controls. Furthermore, in the AGE-induced diabetic microenvironment, ROS levels, mitochondrial dysfunction, excessive accumulation of Fe^2+^ increased, while GPX4 expression decreased. Based on the above results, we hypothesized that the excessive production of AGEs may be the main cause of cell death in the diabetic microenvironment, and ferroptosis may be only one of several potential major molecular mechanisms involved in this process.

In the cellular antioxidant defense system, the KEAP1/NRF2 metabolic pathway is the central regulator of ferroptosis, inhibiting ferroptosis directly by targeting the reductive-oxidative pathway.^20,52^ Curcumin is one of the main bioactive phenolic compounds isolated from *Curcuma longa L*, an ancient medicinal plant with anti-inflammatory, antioxidant, and hypoglycemic properties.^53,54^ We, in this study, found that curcumin can inhibit ferroptosis and promote the osteogenic differentiation of BMSCs by decreasing the excessive production of ROS, reducing Fe^2+^ levels, and activating the KEAP1/NRF2 pathway to upregulate the expression of GPX4 in the diabetic microenvironment. These findings suggest that curcumin could be used to prevent and alleviate DOP.

tFNA, as a multifunctional nanomaterial, has been widely applied in various fields of biomedical science to deliver DNA, RNA, peptides, and small-molecule compounds.^30,55^ More importantly, these nanoparticles have the advantage of good biosafety and high drug utilization. In this study, we developed a ferroptosis-suppressing tFNA nanoparticles to deliver curcumin to BMSCs treated with AGEs and to DOP model mice, to evaluate tFNA-Cur application as a potential treatment option of DOP. Our results demonstrated that ferroptosis-suppressing nanoparticles induced the osteogenic differentiation of BMSCs treated with AGEs by activating the NRF2/GPX4 pathway. Furthermore, our findings showed that these nanoparticles possessed high stability and improved the circulation time of curcumin in physiological fluids, which enhanced its bioavailability, delivering curcumin to the bone marrow and achieving better therapeutic effects at lower doses.

**Scheme 1:**
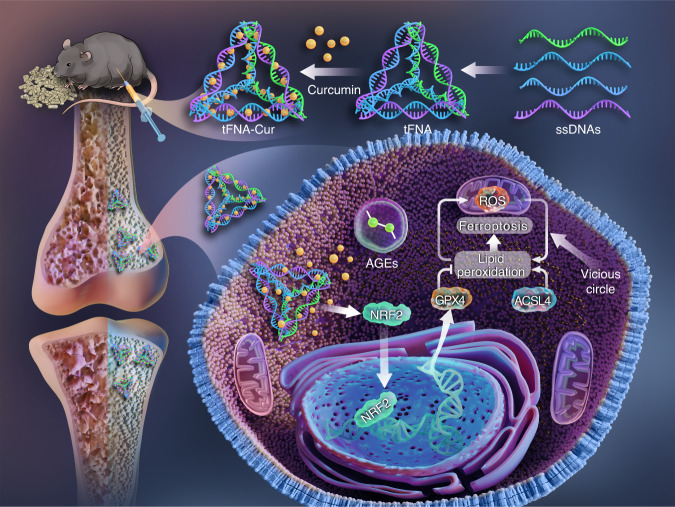
tFNA-Cur inhibits ferroptosis by activating the NRF2/GPX4 pathway, promoting the osteogenic differentiation of BMSCs in diabetic microenvironment, reducing trabecular loss, and increasing bone formation

Targeting ferroptosis to alleviate diabetic osteoporosis is a promising therapeutic approach. In this study, we demonstrated that tFNA-Cur nanoparticles could inhibit ferroptosis, enhance mitochondrial function by activating the NRF2/GPX4 pathway, induce the osteogenic differentiation of BMSCs in the diabetic microenvironment, as well as reduce trabecular loss and increase bone formation in a mouse model of DOP (Scheme 1). These findings suggest that the ferroptosis-suppressing nanoparticles based on DNA tetrahedron technology hold a promising potential for the treatment of DOP and other ferroptosis-related diseases.

However, certain limitations require attention and improvement. Firstly, enhancing the serum stability of ferroptosis-suppressing Nanoparticle remains a priority. While tFNA-Cur has demonstrated more stability in normal serum environments, further improvements in stability would enhance the absorption and effectiveness of drug. Secondly, accurate quantification of natural products carried by frame nucleic acids contributes to clinical transformation. At present, the amounts of natural products carried by frame nucleic acids is uncontrollable, although the synthesis of nanocomposites is simple, it restricts clinical application of nano-ferroptosis inhibitors. Lastly and most importantly, how can the BMSCs be targeted more specifically in vivo? For further research, we linked the bone targeting peptide SDSSD on one of the ssDNA through click chemistry, and achieved bone targeting by encapsulating the BMSCs membrane on the ferroptosis-suppressing nanoparticle.

## Materials and methods

### Fabrication of tFNA‑Cur

Firstly, four single-stranded DNA molecules (ssDNAs, Table S1) (Sangon, Shanghai, China) were mixed in equimolar quantities, dissolved in TM buffer (Tris-HCl and MgCl_2_, pH=8.0), heated at 95 °C for 10 min, and then cooled at 4 °C for 20 min.^56,57^ Secondly, the product of the first step, tFNA (200 nmol/L), was mixed with various concentrations of curcumin (5 μmol/L, 10 μmol/L, 15 μmol/L, 20 μmol/L, 25 μmol/L, and 40 μmol/L) and oscillated for 3 h.

### Characterization of tFNA‑Cur

The synthesis of tFNA-Cur was assessed using PAGE (polyacrylamide gel electrophoresis). The Zeta potentials and sizes of tFNA-Cur and tFNA were measured using DLS (dynamic light scattering) with the Nano ZS instrument (Malvern, UK). The shapes and sizes of tFNA-Cur were determined using TEM (transmission electron microscopy) with the Libra200 microscope (Zeiss, Oberkochen, Germany) and AFM (atomic force microscopy) with the Cypher VRS microscope (Oxford Instruments, United Kingdom). The complete spectra of tFNA, curcumin, and tFNA-Cur were analyzed using an ultramicrospectrophotometer (UV5 Nano, Mettler Toledo, Switzerland).

### Release and stability of tFNA-Cur

Based on previous research,^38^ we divided the dialysis bag (30 kD; Solarbio, Beijing, China) into an outer liquid (30 mL) and inner fluid (3 mL) using PBS (pH 7.4, 0.01 mol/L) as the medium. Equimolar concentrations of curcumin and tFNA-Cur (20 μmol/L) were dissolved in the inner fluid at 37 °C with constant agitation (150 r/min). The OD value of the released curcumin in the external liquid was determined using an ultra-microspectrophotometer. To assess the stabilities of tFNA and tFNA-Cur, they were separately incubated with 2% or 10% serum for different time intervals (0 h, 2 h, 4 h, 6 h, 8 h, 10 h, and 12 h). Agarose gel electrophoresis was employed to analyze the stability results. Finally, images of tFNA and tFNA-Cur were captured using an ultraviolet exposure apparatus (Bio-Rad, Hercules, USA).

### Isolation and culture of BMSCs

Male C57 mice, aged 4 weeks, were obtained from GemPharmatech (Jiangsu, China). BMSCs were isolated from the mice’s bone marrow, flushed with-MEM (HYCLONE, Pittsburgh, USA), 10% fetal bovine serum, and 100 U/mL penicillin-streptomycin (HYCLONE, Pittsburgh, USA).^58,59^

### BMSCs uptake of curcumin and tFNA-Cur

BMSCs were cultured in a confocal dish with 10 000 cells and treated with curcumin and tFNA-Cur for 6 and 12 h. Images of the cellular uptake of curcumin and tFNA-Cur were captured using a confocal microscope (Olympus, Tokyo, Japan).

### AGEs-induced ferroptosis and osteogenic differentiation of BMSCs

The second-passage BMSCs were seeded in a 96-well plate (10 000 cells per well) and treated with different concentrations of tFNA-Cur (with curcumin concentrations of 5, 10, 15, 20, and 25 μmol/L, and tFNA at 200 nmol/L) for 12 h. Additionally, they were exposed to AGEs at various concentrations (25, 50, 100, 150, 200, and 250 µg/mL) for 24 h. Subsequently, the BMSCs were incubated with 10% CCK-8 reagent (KeyGEN Biotech) for 30 min at 37 °C, and their optical density was measured at 450 nm. Based on the results from the CCK-8 assay and ALP staining, the concentration of AGEs (Bioss, Beijing, China) at 150 µg/mL was determined to simulate a diabetic microenvironment. The BMSCs were pre-treated with tFNA, curcumin, and tFNA-Cur for 12 h, and then treated with 150 µg/mL of AGEs for 24 h to investigate the suppression of ferroptosis. Likewise, the same pretreatment was conducted, followed by exposure to an osteogenic differentiation medium (containing 10 nmol/L dexamethasone, 50 µg/mL ascorbic acid, and 5 mmol/L β-glycerophosphate) for 7 days, with continuous exposure to 150 µg/mL AGEs.

### ALP and alizarin red staining

After pretreatment with tFNA-Cur for 12 h and subsequent treatment with AGEs (150 µg/mL) for 7 days, BMSCs were fixed with 4% paraformaldehyde (at 4 °C for 20 min) and stained with the ALP Kit (C3250S, Beyotime, China) at 37 °C for 10 min. Similarly, after 14 days, the cells were incubated with Alizarin Red S at room temperature for 5 min. ALP activity and mineralized nodules were observed and photographed under a light microscope.

### ROS, MMP level detection assay

Following the aforementioned treatment, BMSCs were first pretreated with tFNA-Cur for 12 h and then exposed to AGEs for 24 h. Subsequently, the cells were incubated with Hoechst 33342 (1X, C1028, Beyotime, China) for 10 min and DCFH-DA (10 μmol/L, S0033S, Beyotime, China) for 20 min to detect the level of ROS. Additionally, they were incubated with Rhodamine 123 (1X, C2008S, Beyotime, China) for 20 min to assess the level of MMP. After washing with PBS, images of ROS and MMP were acquired using a confocal microscope.

### FerroOrange staining

The intracellular level of Fe^2+^ was assessed using the FerroOrange probe (1 μmol/L, MkBio, Mx4559). Following the specified treatments, similar to the procedure for ROS and MMP assessment, the BMSCs were washed with PBS and then incubated with Hoechst 33342 for 10 min and FerroOrange for 20 min. Subsequently, fluorescence images of the BMSCs were captured using a confocal microscope.

### TEM

Mitochondrial morphology changes were observed using TEM. Following the aforementioned treatment, BMSCs were fixed with a 3% glutaraldehyde solution at 4 °C for 16 h. Subsequently, the BMSCs were dehydrated using acetone, embedded in Epon812, sectioned (60-90 nm), and stained with uranium acetate and lead citrate. Finally, the mitochondrial morphology of the various treatment groups was examined and captured using a JEM-1400FLASH transmission electron microscope.

### Quantitative RT-PCR analysis

The transcriptional levels of *Alp*, *Runx2*, *Osx*, *Opn*, *Gpx4*, *Acsl4*, and *Nrf2* were evaluated using reverse transcription-polymerase chain reaction (RT-PCR). The primer sequences can be found in Table S2 (Tsingke Biotech). Total RNA was extracted using TRIzol reagent (Thermo Fisher Scientific, MA, USA). cDNA synthesis was performed using the SYBR^®^ Premix Ex Taq II (Perfect Real Time kit; Takara, Dalian, China). The target genes were measured using SYBR^®^ Green I master mix in Q7 (ABI QuantStudio 7, Thermo Fisher, USA). The results were analyzed using the 2^−∆∆CT^ relative quantitative method, with *β-Actin* (Table S2) serving as the control gene. All experiments were repeated three times.

### Western blot analysis

The BMSCs were lysed using a cell protein extraction reagent (KEYGEN Biotech, Nanjing, China), and then mixed with loading buffer at a ratio of 4:1 (v/v) before being boiled for 5 min. The proteins were separated using 10% SDS-PAGE and subsequently transferred onto a PVDF membrane. The membrane was then blocked with blocking buffer (Thermo Fisher Scientific, MA, USA) for 10 min and incubated with the following antibodies (Table S3) overnight at 4 °C. On the second day, the bands were incubated with a secondary anti-rabbit antibody (1:3 000 BEYOTIME, Shanghai, China) for 1 h after washing with TBST. The results were visualized using an ECL chemiluminescence detection system (Bio-Rad, Hercules, CA, USA).

### IF staining

BMSCs were fixed using a 4% paraformaldehyde solution (4 °C, 25 min), permeabilized with 0.5% Triton X-100 (room temperature, 20 min), blocked with 5% sheep serum (37 °C, 20 min), and washed with PBS at each step, except for the final step. They were then incubated with specific antibodies: anti-ALP (1:200, HUABio, Zhejiang, China), anti-RUNX2 (1:200, HUABio, Zhejiang, China), anti-OSX (1:200, HUABio, Zhejiang, China), anti-GPX4 (1:200, HUABio, Zhejiang, China), anti-ACSL4 (1:200, HUABio, Zhejiang, China), and anti-NRF2 (1:200, HUABio, Zhejiang, China) overnight at 4 °C. The next day, BMSCs were incubated with a secondary anti-rabbit antibody (1:200, Invitrogen, Carlsbad, USA) at 37 °C for 1 h. The cytoskeleton was stained with phalloidin (37 °C, 20 min), and the Nucleus was stained with DAPI (37 °C, 10 min), followed by cleaning with PBS at each step. Images were captured using a confocal microscope (Olympus, Tokyo, Japan).

### Animal experiments

All animal experiments were conducted with the approval of the Animal Ethics Committee of West China Hospital of Stomatology, Sichuan University. Four-week-old male C57BL/6J mice were purchased from GemPharmatech (Nanjing, China) and raised in pathogen-free conditions at (55 ± 5)% humidity and (24 ± 2)°C. First, type 2 diabetes mellitus was induced according to previously published studies.^52,60^ To investigate the effect of diabetes on bone microstructure, mice were randomly divided into two groups: Control, HFD&STZ (*n* = 6). To assess the therapeutic effects of tFNA, curcumin and tFNA-Cur on DOP, mice were randomly divided into five groups: Control, DOP, DOP+tFNA, DOP+curcumin, and DOP+tFNA-Cur (*n* = 6). Throughout the experiment, the Control group mice were fed an ordinary diet (10% kcal from fat), while the model and treatment groups were fed a HFD (60% kcal from fat). After four weeks, all groups except the control group were injected intraperitoneally with STZ (35 mg/kg) for seven days to induce diabetes. Mice in the Control group received citrate buffer injections. Subsequently, mice with high plasma glucose levels (11.1 mmol/L) accompanied by polyphagia, polydipsia, and polyuria were considered diabetic mice for subsequent experiments. The treatment groups were injected intraperitoneally with tFNA (1 μmol/L, 200 μL), curcumin (40 μmol/L, 200 μL), or tFNA-Cur (tFNA: 1 μmol/L, curcumin: 40 μmol/L, 200 μL) three times weekly for 8 weeks. Furthermore, the Control and DOP groups were injected with saline (0.9%, 200 µL). Body weight and fasting glucose levels were monitored every 2 weeks. Finally, the bones were harvested and analyzed using micro-CT, H&E staining, masson’s trichrome staining, and IF staining for ALP, GPX4, and NRF2.

### Micro-CT analyses

The lower extremities were examined using a SCANCO Medical Micro-Computerized Tomography 50 (70 kV, 200 μA, 300 ms, 10 μm). The regions of interest (ROI) for the femur and tibia were identified at a location 2 mm below the epiphysis.^61^

### AGEs examination in bone and serum

Blood samples were deposited at 4 °C for 30 min and then centrifuged at 3 000 r/min for 10 min to obtain the serum. The levels of AGEs in both the serum and bone samples were determined using a mouse AGEs ELISA kit (YKW-20124, Shanghai, China).

### TUNEL assay

Cell death in the bone tissue was assessed using the TUNEL assay (Beyotime, Shanghai, China). Tissue antigens were retrieved using proteinase K (20 μg/mL, 37 °C, 15 min) and then incubated with TUNEL solution (37 °C, 60 min). Subsequently, the Nucleus was stained with DAPI (37 °C, 4 min). Finally, images were captured using a confocal microscope.

### Histological analysis

The specimens from various treatment groups were fixed with 4% buffered formalin for 72 h. After one month of decalcification, the bones were dehydrated, embedded in paraffin, and sectioned (3 μm thick). The sections were then stained with H&E and masson’s trichrome to visualize tissue morphology and structural components. Additionally, the bone sections underwent IF staining using anti-ALP, anti-GPX4, and anti-NRF2 antibodies as described above-mentioned.

### 3D molecular modeling and docking of curcumin to NRF2

The predicted structures of NRF2 were generated using the Alphafold software. Docking grid documents were created using AutoGrid of Sitemap, and the docking simulation was performed using AutoDock Vina (version 1.2.0). The optimal pose was selected for analyzing the interactions. Finally, the protein-compound interaction figure was generated using PyMOL. In the figure, NRF2 is depicted as a slate cartoon model, while curcumin is shown as cyan sticks, and its binding sites are represented by magenta stick structures.

### Statistical analysis

All experimental data were analyzed using GraphPad Prism 9.3.1 software (San Diego, CA, USA). Comparisons between two groups were conducted using Student’s *t*-test, while comparisons among more than two groups were performed using one-way or two-way ANOVA with Sidak’s multiple comparison test. The experimental results are presented as the mean ± SD values, and *P* < 0.05 was considered statistically significant.

### Supplementary information

SI
