# Supplementary material for: A DNA tetrahedron-based ferroptosis-suppressing nanoparticle: superior delivery of curcumin and alleviation of diabetic osteoporosis
Source: Bone Res. 2024 Feb 29;12:14. doi: 10.1038/s41413-024-00319-7 (PMC10904802; doi:10.1038/s41413-024-00319-7)
Supplement: Supplementary file 1 — SI [file 41413_2024_319_MOESM1_ESM.docx]

Supplementary Information

A DNA Tetrahedron-based Ferroptosis Inhibitor: Superior Delivery of Curcumin and Alleviation of Diabetic Osteoporosis

*Yong Li,^1,2^ Zhengwen Cai,^1^ Wenjuan Ma,^1^ Long Bai,^2^ En Luo,^1, *^ Yunfeng Lin,^1, 3*^*

*^1^ State Key Laboratory of Oral Diseases & National Center for Stomatology & National Clinical Research Center for Oral Diseases, West China Hospital of Stomatology, Sichuan University, Chengdu, Sichuan 610041, P. R. China*

*^2^ Department of Oral and Maxillofacial Surgery, Affiliated Stomatological Hospital, Southwest Medical University, Luzhou, Sichuan 646000, P. R. China*

*^3^ Sichuan Provincial Engineering Research Center of Oral Biomaterials, Chengdu, Sichuan 610041, China*

** Corresponding author: En Luo, Yunfeng Lin*

En Luo.

State Key Laboratory of Oral Diseases & National Center for Stomatology & National Clinical Research Center for Oral Diseases, West China Hospital of Stomatology, Sichuan University, Chengdu, Sichuan 610041, P. R. China

Tel/Fax: 86-28-85503530; E-mail address: luoen521125sina@.com

Yunfeng Lin.

State Key Laboratory of Oral Diseases & National Center for Stomatology & National Clinical Research Center for Oral Diseases, West China Hospital of Stomatology, Sichuan University, Chengdu, Sichuan 610041, P. R. China

Tel: +86-28-85503487; Fax: +86-28-85582167; E-mail address: yunfenglin@scu.edu.cn

**Supplementary Tables**

**Table 1** Sequences of DNA (capital letters denote DNA monomers)

| ss DNA | Base Sequence (5’to 3’) |
| --- | --- |
| S1 | ATTTATCACCCGCCATAGTAGACGTATCACCAGGCAGTTGAGACGAACATTCCTAAGTCTGAA |
| S2 | ACATGCGAGGGTCCAATACCGACGATTACAGCTTGCTACACGATTCAGACTTAGGAATGTTCG |
| S3 | ACTACTATGGCGGGTGATAAAACGTGTAGCAAGCTGTAATCGACGGGAAGAGCATGCCCATCC |
| S4 | ACGGTATTGGACCCTCGCATGACTCAACTGCCTGGTGATACGAGGATGGGCATGCTCTTCCCG |
| Cy5-S1 | Cy5-  ATTTATCACCCGCCATAGTAGACGTATCACCAGGCAGTTGAGACGAACATTCCTAAGTCTGAA |

**Table 2** Sequences of the PCR primers for amplification of expressed genes

| **Genes** | **Sequence(5′→3′)** |
| --- | --- |
| **m*β-Actin*** | F: GACCTGACTGACTACCTCATGAAGAT  R: GTCACACTTCATGATGGAGTTGAAGG |
| **m*Alp*** | F: CCAACTCTTTTGTGCCAGAGA  R: GGCTACATTGGTGTTGAGCTTTT |
| **m*Runx2*** | F: CCGAACTGGTCCGCACCGAC  R: CTTGAAGGCCACGGGCAGGG |
| **m*Osx*** | F: ATGGCGTCCTCTCTGCTTG  R: TGAAAGGTCAGCGTATGGCTT |
| **m*Opn*** | F: GGATTCTGTGGACTCGGATG  R: CGACTGTAGGGACGATTGGA |
| **m*Gpx4*** | F: CTGGGAAATGCCATCAAAT  R: GTCCTTCTCTATCACCTGG |
| **m*Acsl4*** | F: AGCGTTCCTCCAAGTAGA  R: GCCTGTCATTCCAGCAAT |
| **m*Nrf2*** | F: TCTTGGAGTAAGTCGAGAAGTGT  R: GTTGAAACTGAGCGAAAAAGGC |

**Table 3.** Antibodies applied for flow cytometry and immunofluorescence (IF) in this paper

| **Antibodies** | **Company** | **Application** | **Dilution fold** |
| --- | --- | --- | --- |
| Anti-CD29 (FITC) | BioLegend | Flow cytometry | 1:200 |
| Anti-CD44 (FITC) | BioLegend | Flow cytometry | 1:200 |
| Anti-CD45 (APC-Cy7) | BioLegend | Flow cytometry | 1:200 |
| Anti-CD31 (PE) | BioLegend | Flow cytometry | 1:200 |
| Anti-CD34 (PE) | BioLegend | Flow cytometry | 1:200 |
| Anti-CD90 (PE-Cy7) | BioLegend | Flow cytometry | 1:200 |
| RUNX2 | HUABIO | WB; IF | 1:500; 1;200 |
| OSX | HUABIO | WB; IF | 1:500; 1;200 |
| ALP | HUABIO | WB; IF | 1:500; 1;200 |
| OPN | Abcam | WB | 1:1000 |
| β-ACTIN | Abcam | WB | 1:1000 |
| GPX4 | HUABIO | WB; IF | 1:500; 1;200 |
| ACSL4 | HUABIO | WB; IF | 1:500; 1;200 |
| NRF2 | HUABIO | WB; IF | 1:500; 1;200 |
| KEAP1 | Proteintech | WB | 1:1000 |

**Table 4** Molecular docking for NRF2 and Curcumin

| Receptor: NF2L2 (alphafold) | Ligand: CURCUMIN | -7.1 kcal/mol |
| --- | --- | --- |
| Hydrogen bond interaction | GLN26 | 2.3 Å |
|  | ASN467 | 2.2 Å |
| Electrostatic interaction | \ | 2.5Å |
|  |  |  |
| hydrophobic interaction |  | 2.5Å |
|  |  |  |

**
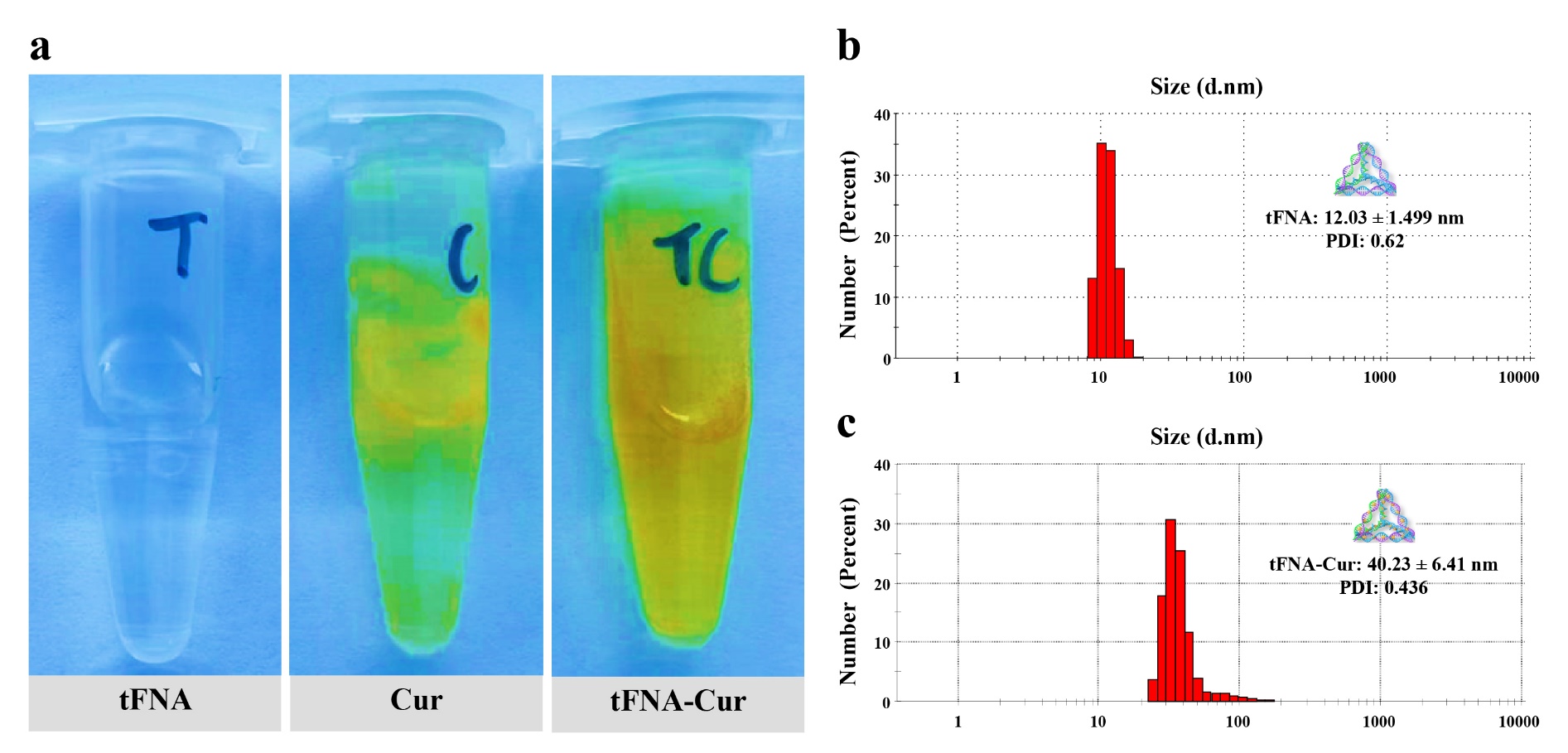
Supplementary Figures**


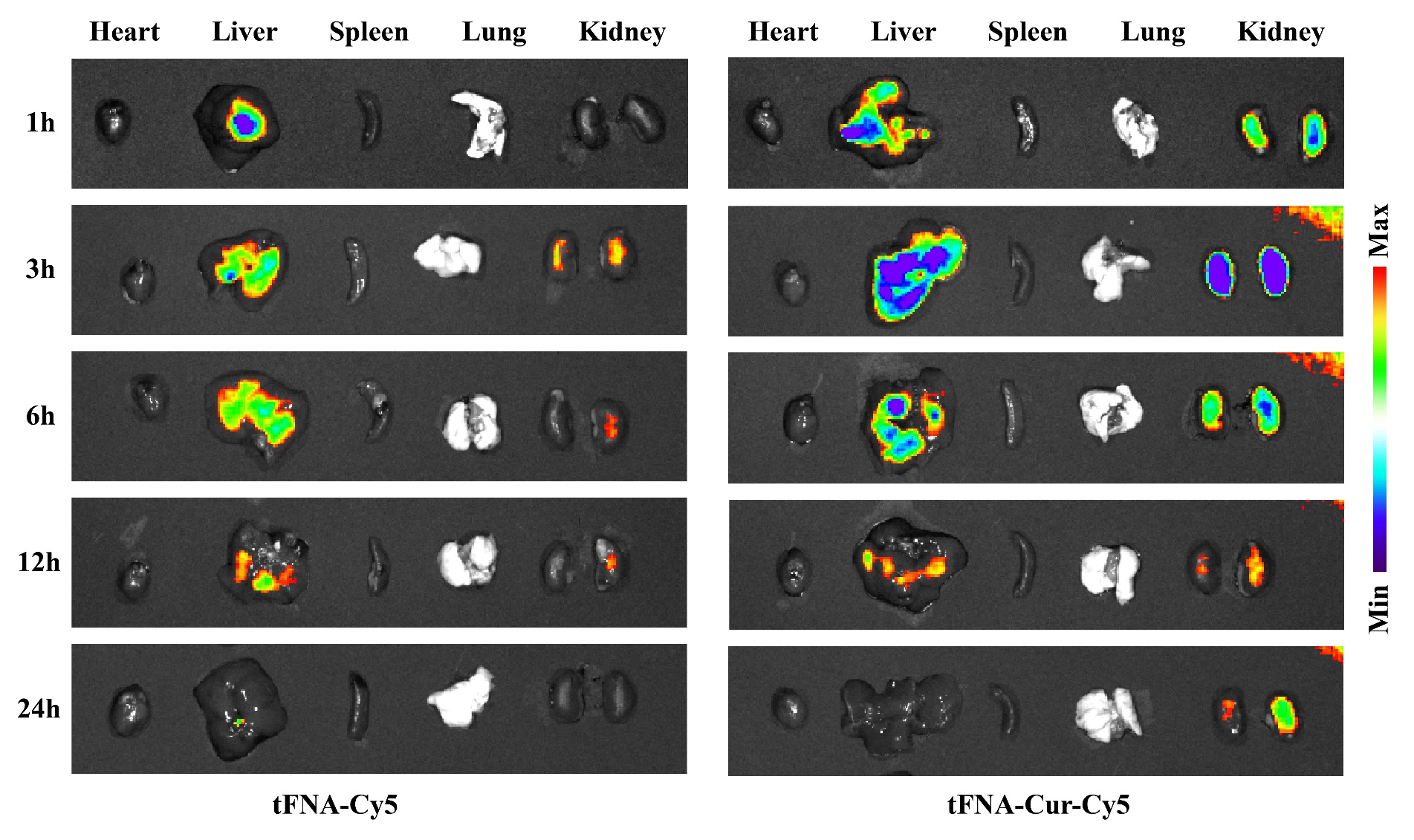
**Fig. 1. a** Images of tFNA, Cur and-tFNA-Cur dissolved in TM buffer. **b** Molecular size of tFNA and tFNA-Cur **(c)**.

**Fig. 2.** In vivo stability evaluation of tFNA and tFNA-Cur labelled with Cy5 in physiological fluids through live imaging of small animals at different time points.


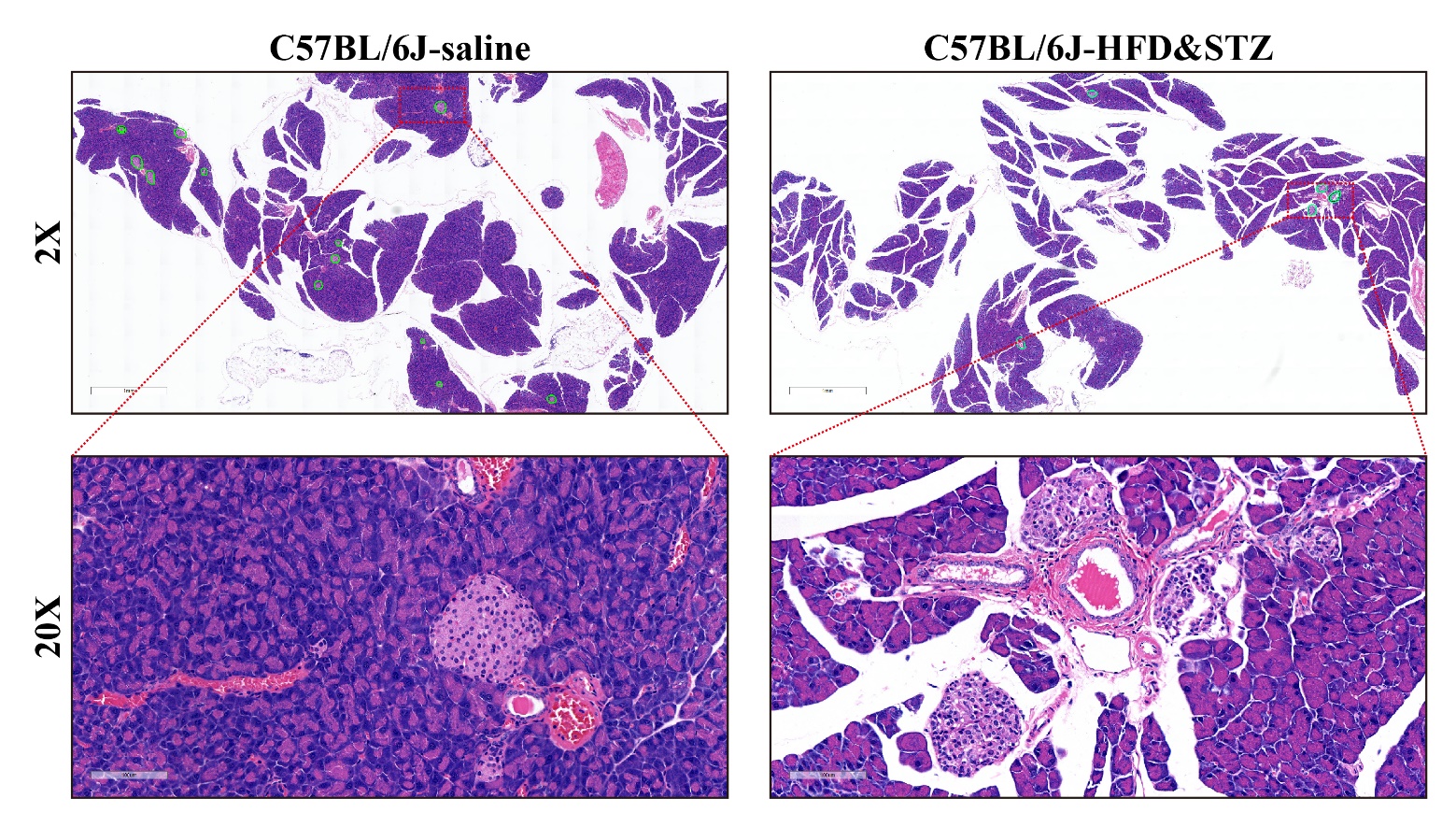
**Fig. 3.** Pancreatic sections of Control and HFD&STZ mice.

**
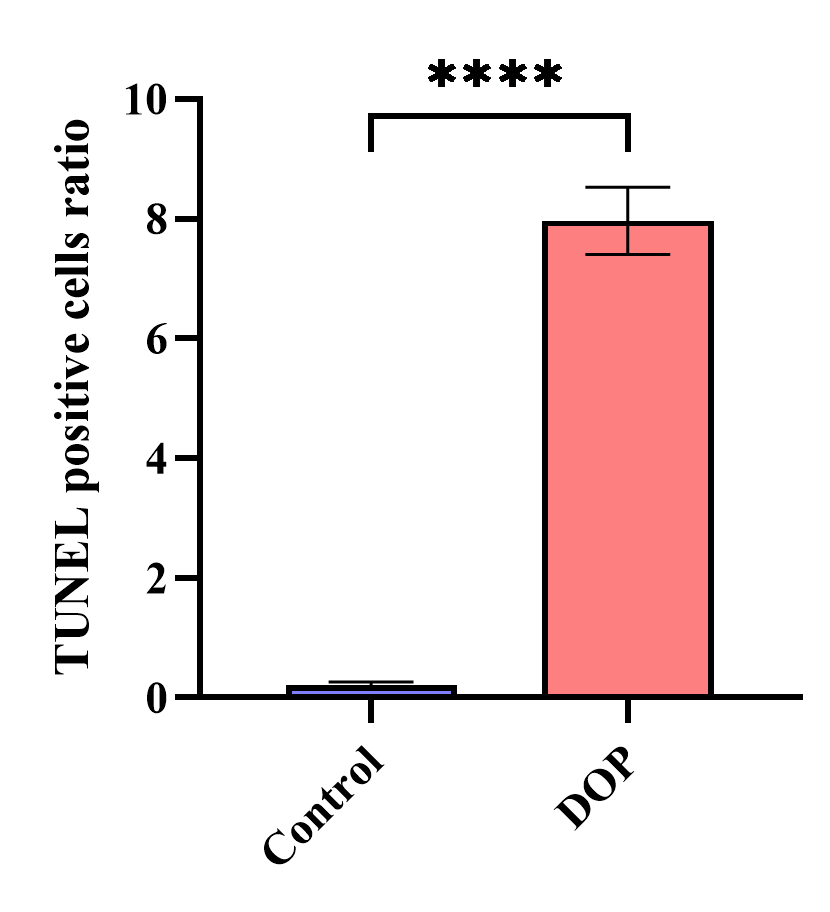
**

**Fig.4.** Semiquantitative analysis of TUNEL-positive cells.


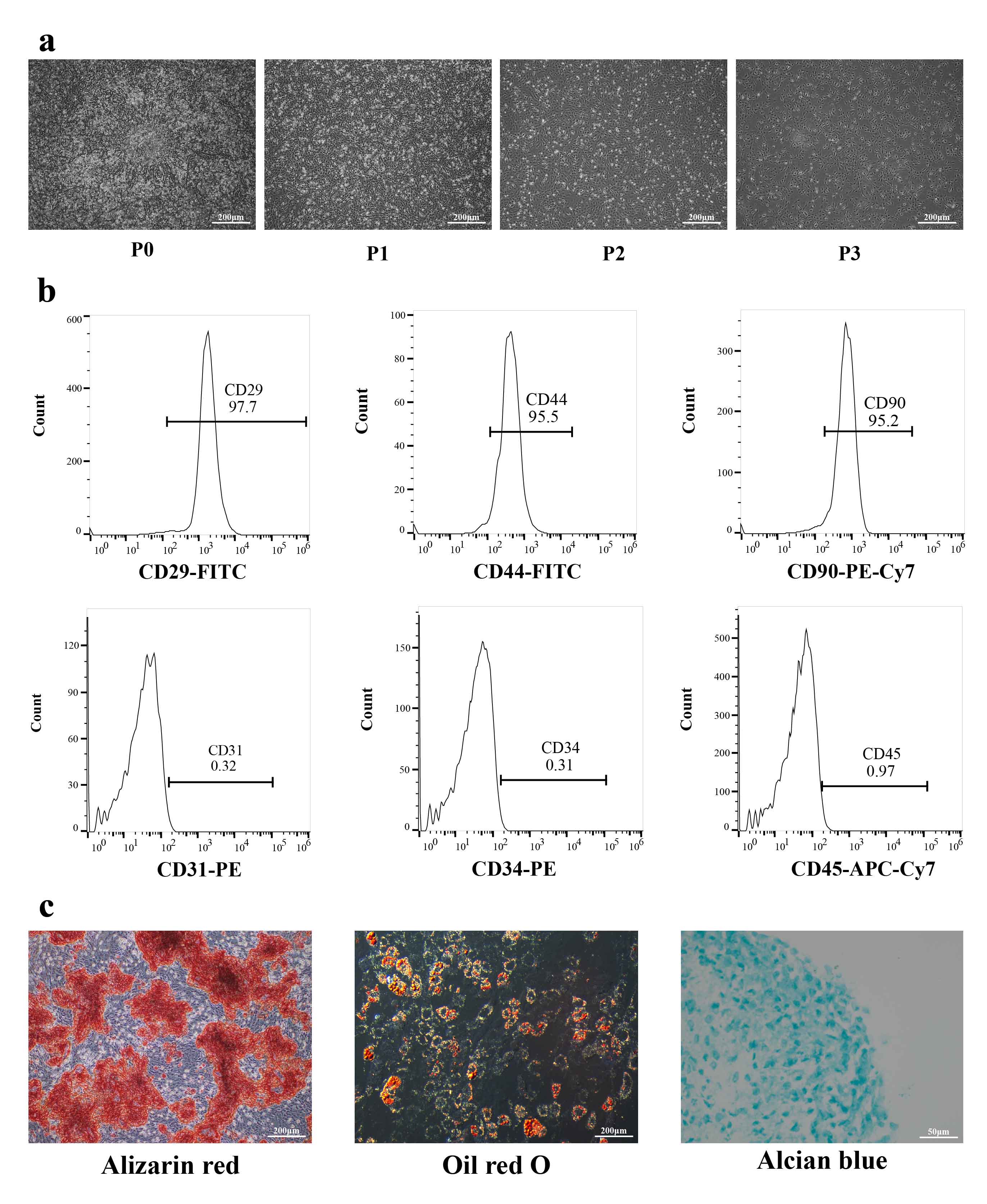
**Fig. 5.** The isolation and culture of BMSCs. **a** Primary cell morphology of BMSCs; **b** Flow cytometry showed the expression of the surface markers CD29, CD44, CD90, CD31, CD34, and CD45 on BMSCs. **c** Osteogenesis, adipogenesis, and chondrogenic differentiation of BMSCs.


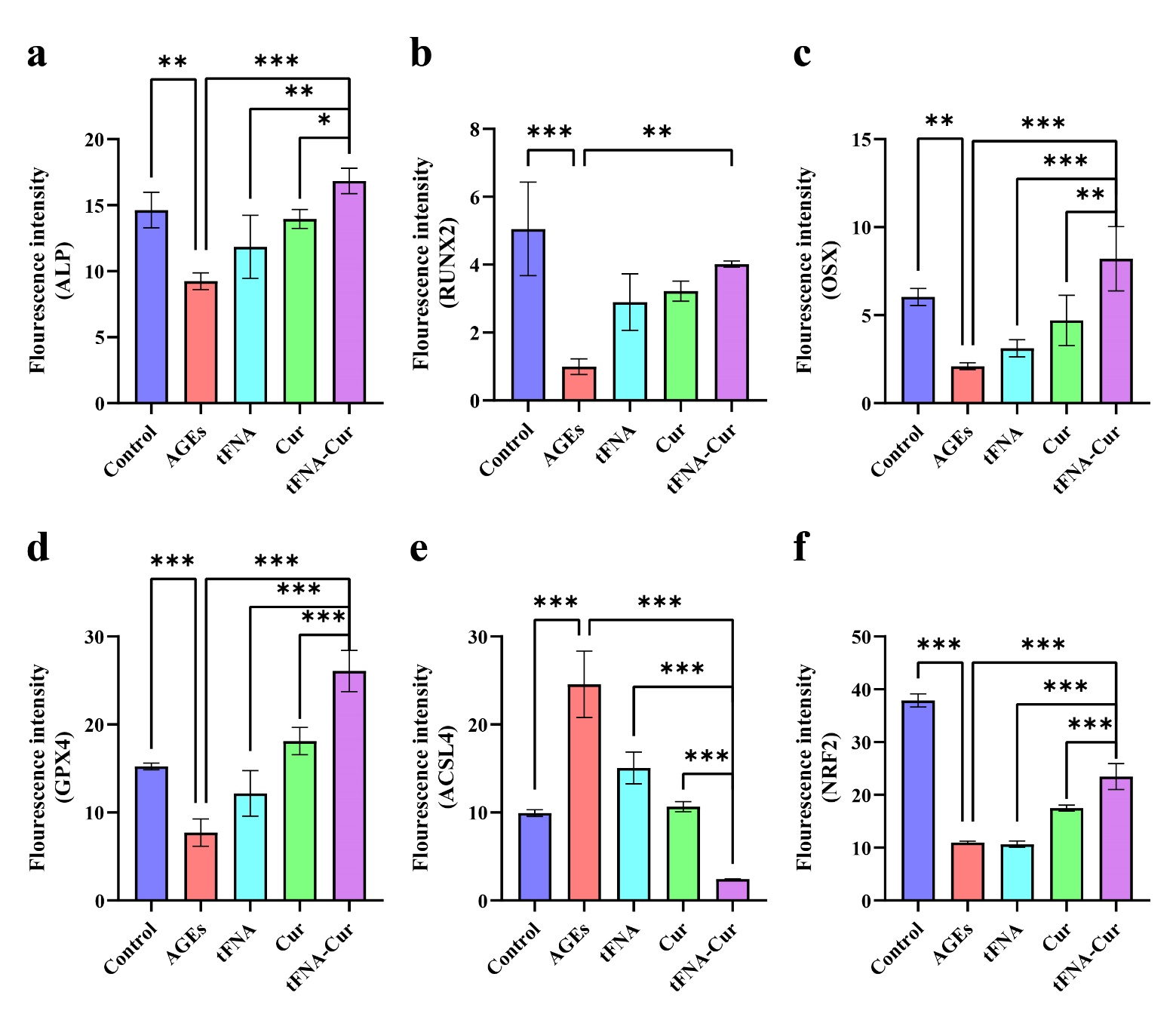

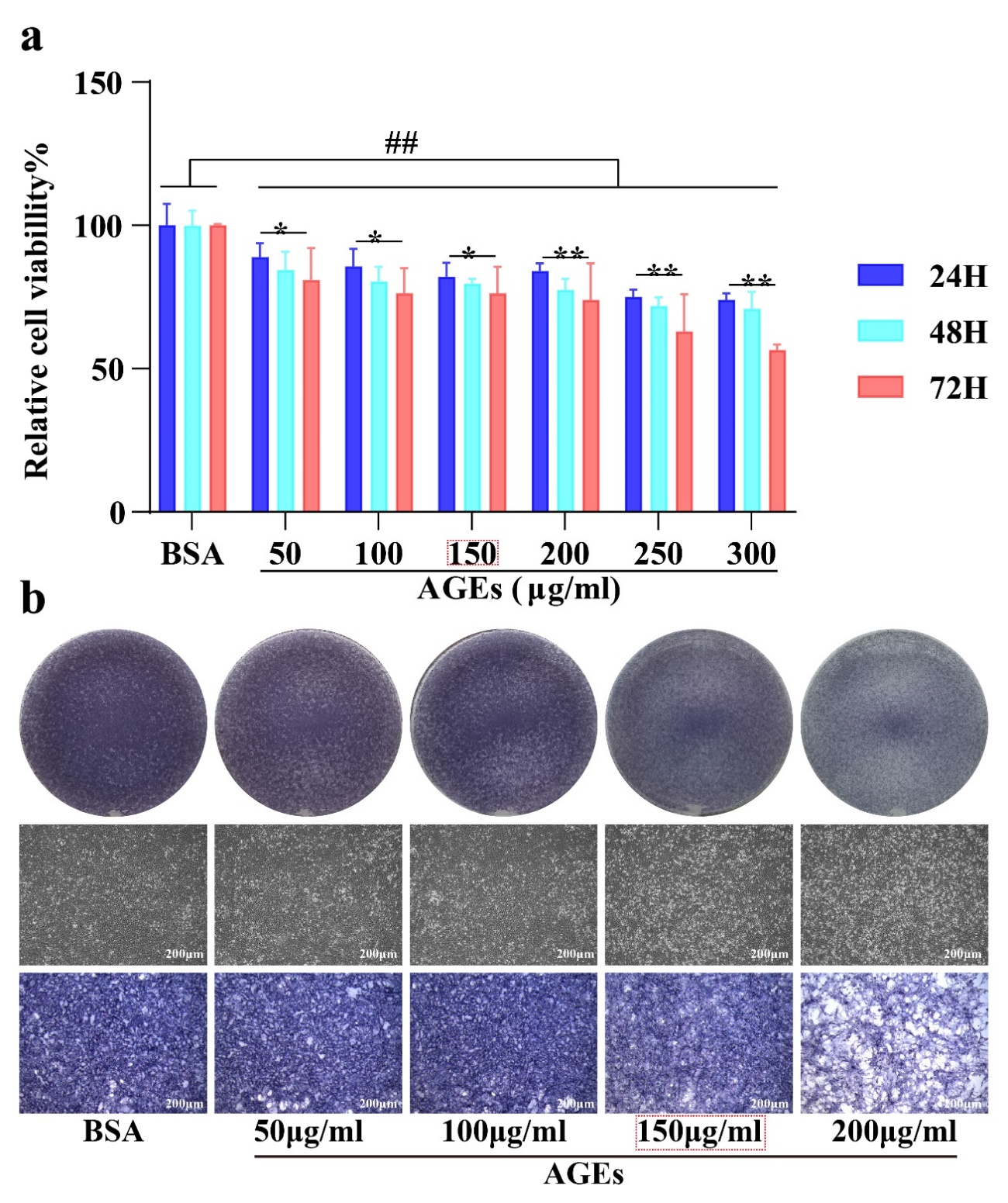
 **Fig. 6. a** BMSCs were treated with AGEs at various concentrations for 24/48/72 h, and cell viability was evaluated using the CCK-8 kit**. b** The appropriate concentration of AGEs by ALP staining.


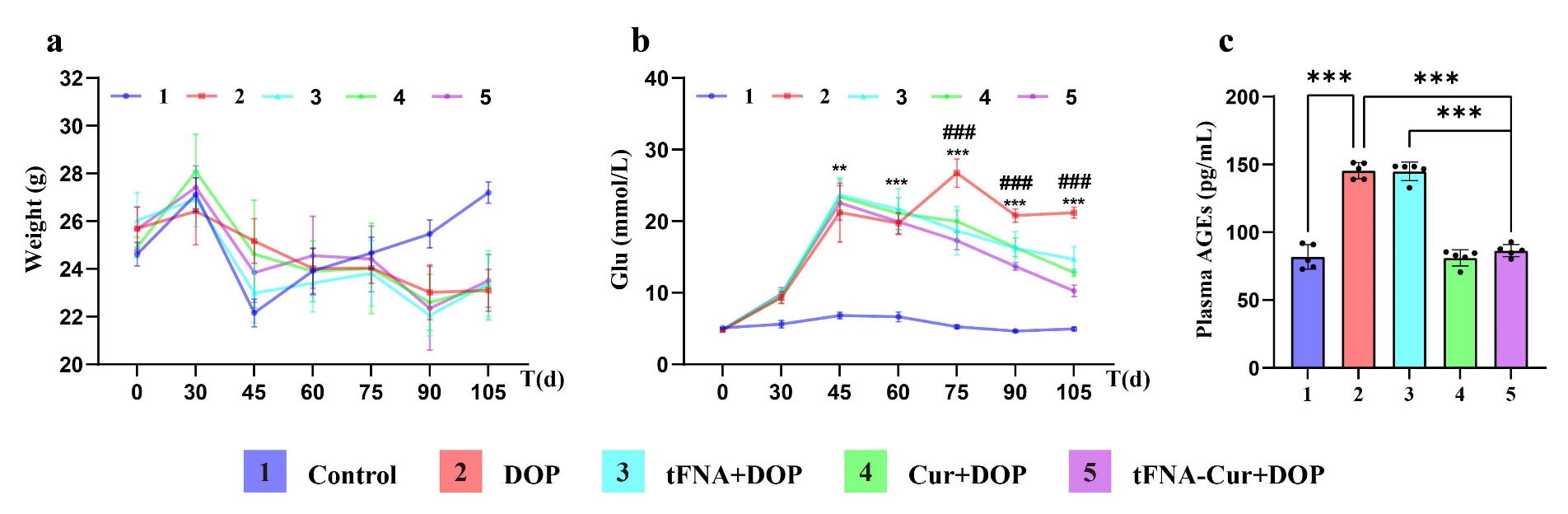
**Fig. 7. (a-c)** Semiquantitative analysis of the fluorescence intensity of ALP, RUNX2 and OSX, **(d-f)** Semiquantitative analysis of the fluorescence intensity of GPX4, ACSL4 and NRF2.

**Fig. 8.** Verification of the DOP mouse model treated by tFNA-Cur. Body weight **a** and blood glucose **b** were assessed biweekly. **c** Plasma AGEs by Elisa. *** p < 0.01 (Ctrol vs DOP),*** p < 0.001,^##^ p < 0.01 (DOP vs tFNA-Cur+DOP),^###^ p < 0.001.* Each group contained 6 mice.


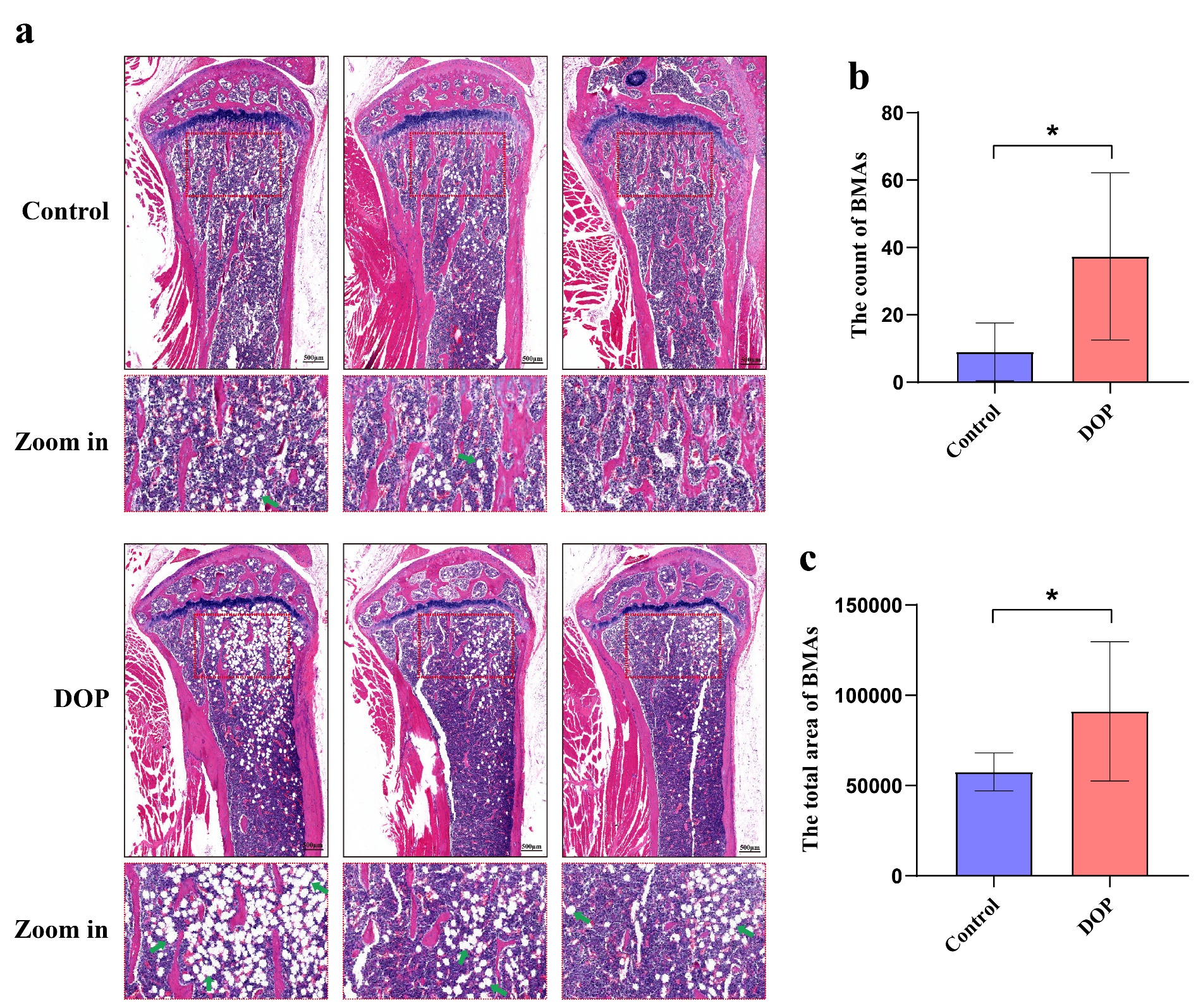
**Fig. 9. a** Images of H&E staining in distribution of bone marrow adipocytes (BMAs) in the tibial bone marrow of the control and DOP. Scale bar: 500 μm. Green Arrow: BMAs. The Count (**b**) and the total area (**c**) of BMAs. Data is presented as mean ± SD (n= 3). Statistical analysis: *p< 0.05.


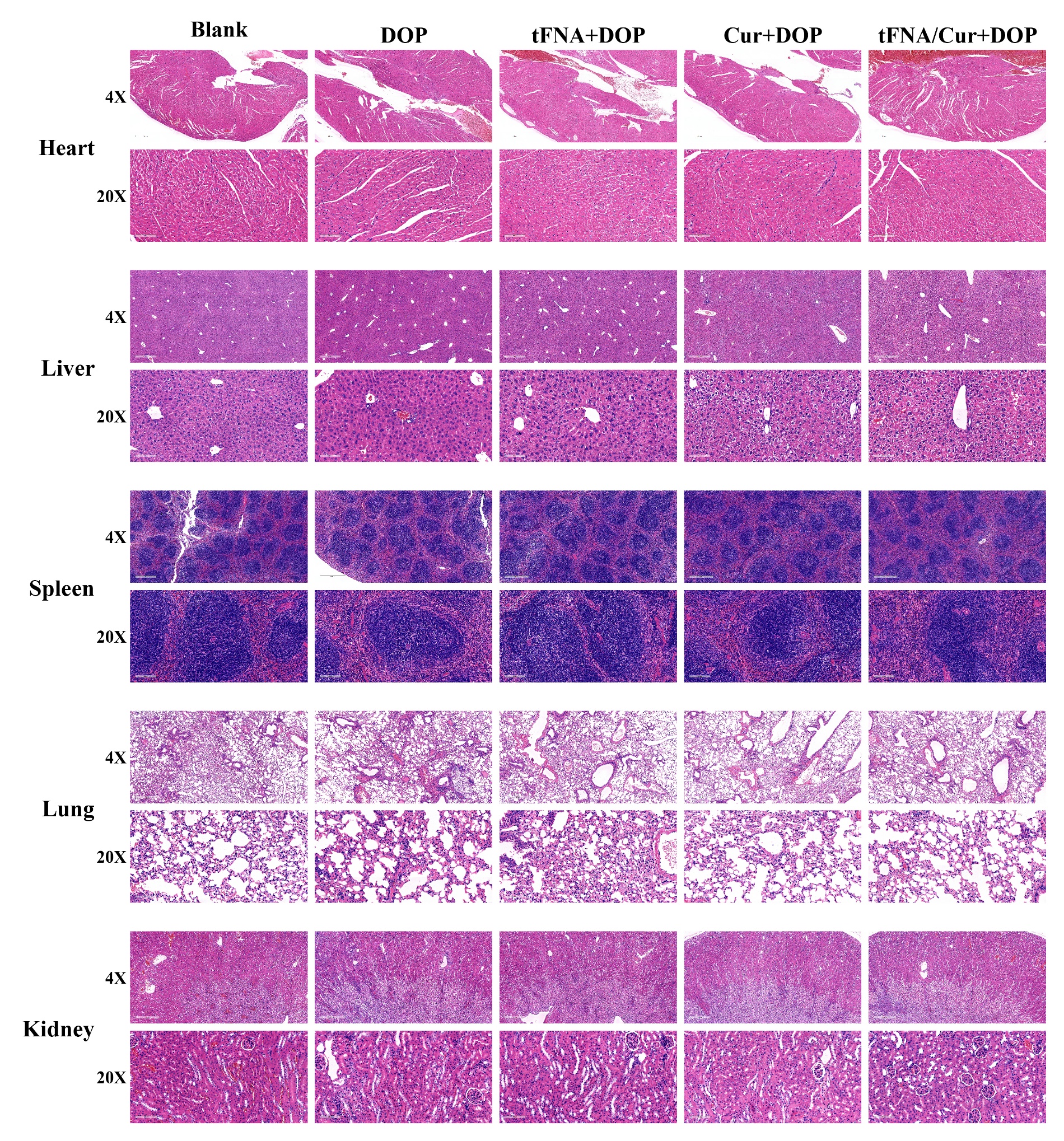


**Fig. 10.** tFNA-Cur targeted ferroptosis alleviates diabetic osteoporosis in vivo without obvious toxicity to the organs of the animals.
